# Colon Histophysiological Features and Gut Microbiome in Tolerant and Susceptible to Oxygen Deficiency Wistar Rats After the Prolonged Intermittent Hypoxic Exposure

**DOI:** 10.3390/biom16070935

**Published:** 2026-06-23

**Authors:** Maria Kirillova, Dzhuliia Dzhalilova, Natalia Zolotova, Vladimir Kirillov, Larisa Ogneva, Mikhail Kirillov, Tatiana Portnova, Natalia Berlizeva, Nikolai Fokichev, Olga Makarova

**Affiliations:** 1 Laboratory of Immunomorphology of Inflammation, Petrovsky National Research Center for Surgery, Moscow 117418, Russia; marusyasilina99@yandex.ru (M.K.); natashazltv@gmail.com (N.Z.); portnova.tatiana102@mail.ru (T.P.); berlizeva.ns@yandex.ru (N.B.); fokichev.1993@mail.ru (N.F.);; 2 Department of Histology, Petrovsky Medical University, Moscow 119435, Russia; 3 Laboratory of Molecular Genetics, National Medical Research Center for Obstetrics, Gynecology and Perinatology Named After Academician V. I. Kulakov of Ministry of Health of Russian Federation, Moscow 117997, Russia; v.kirillov@dna-technology.ru; 4 DNA-Technology Ltd., Moscow 117587, Russia; ogneva@dna-technology.ru (L.O.); kirillov@dna-technology.ru (M.K.); 5 Faculty of Biology and Biotechnology, National Research University Higher School of Economics, Moscow 117418, Russia

**Keywords:** tolerance to hypoxia, microbiome, hypoxic load, histophysiology, gut

## Abstract

Systemic hypoxia influences the state of the intestinal epithelial barrier and the microbiome; however, the role of the initial tolerance of the organism to oxygen deficiency in the development of these changes remains poorly studied. The aim of the study was to evaluate the colon histophysiological features and the gut microbiome in rats that were tolerant and susceptible to hypoxia under intermittent hypoxic exposure of varying severity. In male Wistar rats, tolerance to oxygen deficiency was determined according to the *Hif1a*, *Epas1*, and *Hif3a* expression levels in peripheral blood leukocytes, after which they were subjected to intermittent hypoxic exposure at an “altitude” of 5000 m or 7000 m for 1 h daily for 21 days. Subsequently, the state of the intestinal epithelial barrier was assessed using histological, histochemical, and immunohistochemical methods, and the microbiota composition was analyzed by PCR. Under normoxic conditions, in comparison with rats that are tolerant to hypoxia, susceptible animals demonstrated a greater volume fraction of goblet cells and a low abundance of *Parabacteroides* spp. Intermittent hypoxic exposure induced multidirectional changes depending on the initial tolerance and the severity of the regimen. In tolerant-to-hypoxia animals, an increase in the goblet cells volume fraction was detected after the exposure at the 5000 m “altitude”, while at an “altitude” of 7000 m, a decrease in the number of cells in the lamina propria of the mucosa and *Clostridium perfringens gr*. abundance, as well as a reduction in the *Firmicutes/Bacteroidetes* ratio, was observed. In susceptible-to-hypoxia animals, a higher abundance of *Clostridium perfringens gr*. in comparison with tolerant rats was revealed after the exposure at an “altitude” of 7000 m, with no structural changes in the intestinal wall. Thus, intermittent hypoxic exposure led to a rearrangement of the gut microbiome and the morphofunctional characteristics of the intestinal barrier, and the severity of these changes depended on the initial tolerance of the organism to oxygen deficiency and the severity of the hypoxic regime, which should be taken into account when conducting biomedical research.

## 1. Introduction

The structural and functional integrity of the colon epithelium plays a key role in ensuring barrier function, digestive processes, nutrient absorption, and maintaining immune and metabolic homeostasis [1]. It is known that systemic hypoxia, developing under high-altitude conditions, can lead to ischemia of the colonic mucosa, damage to the intestinal barrier, and dysbiotic changes in the microbiota [2]. According to the literature data, alpinists who participated in a 47-day expedition to the Himalayas (staying at an altitude of more than 5000 m for 29 days) demonstrated significant changes in the fecal microbiota composition—the abundance of beneficial bacteria, including *Bifidobacterium*, *Atopobium*, *Coriobacterium*, and *Eggerthella lenta*, decreased, while the abundance of opportunistic bacteria, such as γ-proteobacteria and specific enterobacteria (e.g., *Escherichia coli*), increased in comparison with the indicators at sea level 14 days before the expedition and 14 days after it [3]. Furthermore, Qi P et al. (2023) [4] established that hypoxemia caused by acute high-altitude hypoxia exposure led to changes in the human microbiome. When people moved from Lanzhou (altitude 1500 m) to Gannan (altitude 3000 m), less than 48 h after arrival, they exhibited symptoms of hypoxemia—oxygen saturation below 95%. The microbiome study revealed changes in the relative abundance of bacteria of different genera, the level of tyrosine secreted by them, and the erythropoietin content in peripheral blood [4]. It was demonstrated that an increase in the relative abundance of *Bacteroides*, *Parabacteroides*, and *Odoribacter*, combined with a decrease in the relative abundance of *Saccharibactria_Genera_incertae_Sedis*, led to an increase in tyrosine level, and its absorption into the blood stimulated erythropoietin production in people with hypoxemia caused by acute high-altitude hypoxia exposure. Erythropoietin, in turn, played a key role in adaptation to oxygen deficiency—it activated erythropoiesis, which increased the blood oxygen transport function [5].

In addition, some gut microbiome representatives synthesize short-chain fatty acids, including butyrate, propionate, and acetate [6], which are a source of energy for colon epithelial cells [7]. Kelly C. J. et al. (2015) demonstrated that switching from glucose oxidation to butyrate oxidation during its excess led to increased oxygen consumption and, as a consequence, to metabolic local hypoxia and stabilization of the main response regulator to oxygen deficiency—the HIF-1α (Hypoxia-Inducible Factor) protein [8]. At the same time, a decrease in the number of butyrate-producing bacteria led to a reverse metabolic switch and oxygen accumulation in intestinal epithelial cells, which freely diffused into the lumen and promoted the proliferation of opportunistic aerobic bacteria [9,10]. In experimental studies on colitis models induced by 2,4,6-trinitrobenzenesulfonic acid and oxazolone, a more severe disease course was revealed in mice with *Hif1a* knockout in colon epithelial cells in comparison with wild-type animals [11]. When studying the therapeutic activity of prolyl hydroxylase inhibitors regulating HIF-1α on an ulcerative colitis model induced by dextran sulfate sodium, it was demonstrated that two of them—Dimethyloxallyl Glycine (DMOG) and FG-4497—reduced the inflammation severity, which confirmed the anti-inflammatory effect of HIF-1α [12]. At the molecular level, this could be associated with the ability of this protein to limit NF-κB transcriptional activity, the main inflammatory response regulator, through IKK- and CDK6 (Cell Division Protein Kinase 6)-dependent pathways, which was demonstrated under inflammatory conditions in vitro and in vivo [13]. Furthermore, under hypoxic conditions, HIF-1α stabilization induced gene expression and synthesis of the membrane protein claudin-1 CLDN1, JAM-A (Junctional Adhesion Molecule-A) protein, and occludin, which maintained the structure and function of tight junctions between colonocytes [14,15]. The mucus layer, formed by goblet cell secretion in the gut, forms a barrier limiting direct pathogen contact with intestinal epithelial cells [16]. Its main components were mucins, among which MUC2 predominated [17]. It was demonstrated that in intestinal epithelial cells, HIF-1α activation led to an increase in MUC2, MUC3, and ITF (Intestinal Trefoil Factor) protein content, which contributed to strengthening the mucus layer barrier function [18,19,20].

In experiments on laboratory animals, microbiome changes under hypoxic exposure were also revealed. Thus, in albino rats subjected to hypobaric hypoxia at an “altitude” of 4872.9 m for 30 days, 8 h daily, a change in the total bacteria number ratio with a predominance of anaerobes and an increase in the number of pathogenic microorganisms were detected [21]. At the same time, in the colonic lamina propria of the mucosa, lymphoid infiltration was revealed, accompanied by a decrease in the number of goblet cells. In a study by Han Y. et al. (2022), male Sprague–Dawley rats were subjected to hypobaric hypoxia for 5 weeks at an “altitude” of 5500 m, after which the animals were monitored for another 3 weeks [22]. Thus, in the first 3 days of hypoxic exposure, *Firmicutes* predominated over *Bacteroidetes*, while at week 5, the opposite was observed. At the genus level, it was demonstrated that *Prevotella* abundance significantly increased after the start of hypoxic exposure, while *Lactobacillus* abundance increased after its termination. During hypoxic exposure, it is necessary to determine the initial tolerance to oxygen deficiency, which influences the severity of inflammatory bowel diseases [23,24,25,26]; however, it was not taken into account in these studies. Resistance to hypoxia is determined by physiological, epigenetic and genetic characteristics [27,28,29]. Tolerant to hypoxia, rats demonstrate higher physical endurance and a 15% longer lifespan compared with susceptible rats, indicating that both parameters are closely linked to the ability to withstand oxygen deficiency [30]. Moreover, it was demonstrated that animals tolerant to hypoxia are characterized by lower blood flow in skeletal muscles and higher blood flow in the brain, heart, kidneys and lungs compared to susceptible rats [31]. In addition, tolerant-to-hypoxia animals are characterized by a high content of mitochondria with a denser packing of cristae and a darker matrix, a large number of small, functionally more active mitochondria and a higher concentration of mitochondrial enzymes compared to susceptible [32,33]. Moreover, susceptible to oxygen deficiency rats exhibited significantly greater oxidative stress, as evidenced by 8-fold higher cardiac malondialdehyde and a more pronounced rise in plasma carbonylated proteins following hypoxic exposure, compared to tolerant rats [34,35]. We previously demonstrated that in response to cold stress, tolerant-to-hypoxia mice demonstrated an increase in the goblet cell volume fraction, while susceptible-to-hypoxia mice exhibited an increase in the number of cells in the lamina propria [36]. Furthermore, susceptible-to-hypoxia organisms were characterized by a more severe course of acute and chronic ulcerative colitis and colitis-associated colorectal cancer [23,24,25,26]. At the same time, their course severity correlated with a high *Hif1a* expression level in the liver and the distal colon. Collectively, these data indicated that the initial organism tolerance to oxygen deficiency could influence the histophysiological features of the colon epithelial barrier, but data on the relationship between these changes and the microbiome were not presented in the literature.

The standard method for determining tolerance to oxygen deficiency is exposure in a decompression chamber at a critical “altitude”, which could lead to pathological and inflammatory changes in internal organs [37,38,39]. We previously developed a safe method for determining tolerance to oxygen deficiency according to the *Hif1a*, *Epas1*, and *Hif3a* expression levels in peripheral blood leukocytes [40]. In this work, for the first time, tolerance to oxygen deficiency was determined by a new method, allowing the animals not to be subjected to a sublethal hypoxic load.

The aim of the work was to identify the histophysiological features of the colon and evaluate the gut microbiome in tolerant and susceptible to oxygen deficiency animals under prolonged intermittent hypoxic exposure of varying severity.

## 2. Materials and Methods

### 2.1. Animals

The study was performed on male Wistar rats aged 2–3 months (*n* = 66), with body weight 250–300 g, obtained from the Stolbovaya branch of the Scientific Center for Biomedical Technologies of the Federal Medical and Biological Agency of Russia. The experimental unit was a single animal. Rats were housed five per 48 × 37.5 × 21 cm cage with a footprint of 1500 cm^2^ (Tecniplast, Buguggiate VA, Italy), which are designed to hold up to 8 rats weighing 200–300 g or less at the regulated room temperature 25 ± 2 °C under 12:12 h light–dark cycle and 40–50% relative humidity with unlimited access to water and food (Char; JSC Range-Agro, Sergiev Posad, Russia). When working with experimental animals, we were guided by the principles of the European Convention for the Protection of Vertebrate Animals used for Experimental and Other Scientific Purposes (Strasbourg, 1986) and Directive 2010/63/EU of the European Parliament and of the Council. The local ethics committee of Petrovsky National Research Center of Surgery approved the study (Protocol No. 11 dated 20 December 2024). All procedures were in accordance with the ‘Animal Research Reporting of In Vivo Experiments’ (ARRIVE) guidelines. According to the literature, hypoxia tolerance depends on an animal’s sex and age. It was demonstrated that hypoxia tolerant organisms predominantly were detected among females, whereas susceptible and normal organisms were predominantly detected among males, and there were sex differences in the morphofunctional state of the immune system [41,42]. In addition, it has been shown that newborn animals are the most tolerant to hypoxia, while prepubertal animals are the least [43]. To equalize sex and age differences, this study was performed only on male rats. Rats were randomly divided into the following experimental groups with a minimum of five animals each.

### 2.2. Hypoxia Tolerance Determined by Real-Time PCR

To determine tolerance to oxygen deficiency in animals not subjected to hypoxic load, 1 mL of blood was obtained from the tail vein under intramuscular zoletil anesthesia (2 mg/kg; Virbac Sante Animale, Carros, France) into tubes with K3-EDTA (Greiner Bio-One, Kremsmünster, Austria), erythrocytes were lysed, and IntactRNA (Evrogen, Moscow, Russia) was added for RNA fixation. Subsequently, total RNA was isolated from leukocytes using the RNASolo kit (Evrogen), and reverse transcription was performed using the MMLV RT Kit (Evrogen). The mRNA *Hif1a*, *Epas1*, and *Hif3a* expression in peripheral blood leukocytes was determined using qPCRmix-HS SYBR (Evrogen) and primers synthesized by Evrogen [31], by real-time PCR relative to *Gapdh* expression on a DTprime amplifier (DNA-Technology, Moscow, Russia). The relative mRNA concentration of the indicated genes was calculated by ΔΔCq [44].

According to the results of the *Hif1a*, *Epas1*, and *Hif3a* expression levels study in peripheral blood leukocytes, 3 groups of animals were identified—with high (tolerant, *n* = 17), moderate (*n* = 33), and low (susceptible, *n* = 16) tolerance to oxygen deficiency (Table 1).

Moderate to severely hypoxic animals were excluded from the experiment to study the effects of oxygen deficiency on the intestinal barrier and microbiome in animals of the polar groups (tolerant and susceptible to hypoxia) in order to identify a risk group for a severe course of inflammatory bowel diseases.

For each animal, different researchers were involved as follows: a first researcher (M.K.) determined the hypoxia tolerance by real-time PCR and modeled prolonged interval hypoxia (this researcher was the only person aware of the group allocation), the second and the third (T.P. and N.B.) was responsible for animal’s sacrificed from the experiment, the fourth (N.Z.) performed morphological and morphometric study and immunohistochemistry, the fifth and sixth (V.K. and L.O.) analyzed the microbiome.

### 2.3. The Prolonged Intermittent Hypoxia Modeling

**Figure 1:**
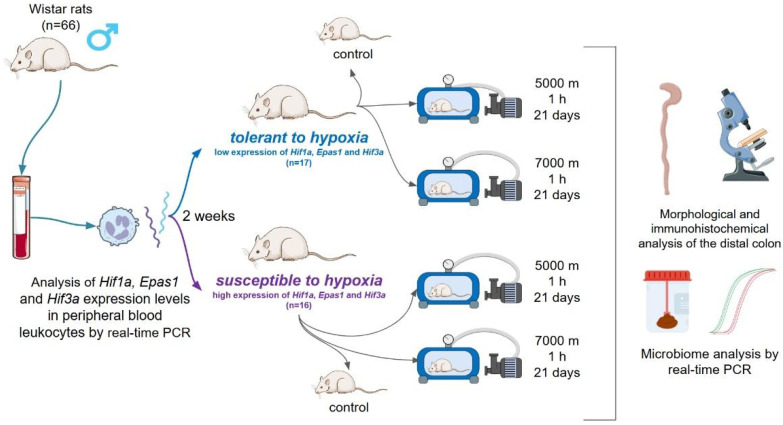
The scheme of the experiment. At a simulated altitude of 5000 m, the partial pressure of oxygen is 85.1 mm Hg, and the oxygen content in the equivalent gas mixture is 11.2%. At a simulated altitude of 7000 m, the partial pressure of oxygen is 64.6 mm Hg, and the oxygen content in the equivalent gas mixture is 8.5%.

Two weeks after blood collection for determining tolerance to oxygen deficiency, some animals were subjected to a hypoxic load at “altitudes” of 5000 m (*n*_tolerant_ = 6, *n*_susceptible_ = 5) and 7000 m (*n*_tolerant_ = 6, *n*_susceptible_ = 6) for 1 h daily for 21 days (Figure 1). The speed of ascent to the simulated altitude was 80 m/s. According to F.Z. Meerson, it was by the 21st day that either long-term adaptation (an increase in tolerance and homeostasis stabilization at a new level) or disadaptation (reserve exhaustion, a drop in functional indicators, organ damage) was formed [45]. The selected hypoxic load regime was the most optimal, since it did not lead to pronounced pathological changes, as was the case with chronic exposure [46,47]. On the 22nd day, the animals were withdrawn from the experiment together with the control group (*n*_tolerant_ = 5, *n*_susceptible_ = 5) by intramuscular administration of zoletil at a dose of 50 mg/kg (Virbac Sante Animale). During the experiment, animal mortality was observed—under hypoxic load at an “altitude” of 7000 m, 1 animal from each group died; at the same time, the susceptible-to-hypoxia rat died on the 1st day of exposure, while the tolerant-to-hypoxia rat died on the 6th day. The deceased animals were excluded from further study.

### 2.4. Sample Collection

On the 21st day, immediately after hypoxic exposure, 1–3 g of feces were collected from the animals, after which their suspension was prepared in sterile saline solution (1 mL) and glycerol (150 µL). A 1.5 cm segment of the distal colon was excised and submerged in 10% neutral buffered formalin (Biovitrum, Saint Petersburg, Russia) for 24 h of fixation for further morphological, histochemical, and immunohistochemical studies.

### 2.5. Preparation of Histological Specimens

The fixed segment of the distal colon was then rinsed under tap water and preserved in 70% ethanol until further use. Following standard histological processing and embedding in Histomix, 5 μm-thick longitudinal sections were prepared. The resultant sections were subjected to various staining protocols—hematoxylin and eosin (H&E), Alcian blue pH 1.0 (targeting highly sulfated mucins), a Periodic Acid-Schiff (PAS) reaction (for neutral mucins), and immunohistochemistry using antibodies against HIF-1α.

### 2.6. Severity of Inflammatory Infiltration

For the number of cells in the lamina propria evaluation, hematoxylin and eosin-stained specimens were photographed using an Axioplan 2 Imaging microscope (Carl Zeiss, Oberkochen, Germany) at 200× magnification in 5 fields of view. The lamina propria connective tissue area was measured, the number of cells located in this zone was counted, and the number of cellular elements per 1 μm^2^ of the lamina propria area was calculated.

### 2.7. Histochemistry and Goblet Cell Count

Mucin terminal carbohydrate groups were either unmodified or acid-modified. The PAS reaction identifies unmodified groups: periodic acid oxidizes vicinal diols into dialdehydes, which subsequently react with the colorless Schiff’s reagent. Conversely, Alcian blue acts as a basic dye that attaches to highly sulfated mucins (strong acids) when the pH is adjusted to 1.0. To assess the goblet cell volume fraction, sections with the PAS reaction were photographed at 160× magnification using an Axioplan 2 Imaging microscope (Carl Zeiss, Oberkochen, Germany) in 5 fields of view under identical lighting conditions. On the histological photographs, using QuPath software (v.0.7.0) [48], an area with correctly oriented crypts from the muscularis mucosae to the lumen was outlined, and its area and the goblet cell area were determined. Each field of view used for cell count analysis included a mucosal area of 0.15–0.25 mm^2^ (from the lumen to the muscularis). The goblet cell volume fraction was calculated as the ratio of their area to the total mucosal area (PAS reaction images). Average brightness measurements were taken for both the goblet cells themselves and the image background (areas without tissue). The goblet cell optical density was derived by taking the logarithm of the average background brightness to average goblet cell brightness ratio. Elevated optical density readings correlate directly with increased content of highly sulfated (Alcian blue) or neutral (PAS-reaction) mucins.

### 2.8. Immunohistochemistry

Immunohistochemical HIF-1α and CD68 detection was carried out by the sandwich method with immunostainer (Bond™-maX, Leica Biosystems, Nussloch, Germany). Colon sections were deparaffinized, citrate buffer pH 6.0 with 0.5% Tween-20 at 100 °C was used for the antigen retrieval and blocked in phosphate-buffered saline with 0.1% bovine serum albumin at room temperature before exposure to antibodies (primary: rabbit polyclonal antibody to HIF-1α, AF1009, Affinity Biosciences, Cincinnati, OH, USA and rabbit recombinant multiclonal antibody to CD68, ab303565, Abcam Inc., Cambridge, UK; secondary: HRP-Linked Caprine Anti-Rabbit IgG Polyclonal Antibody SAA544Rb19, Cloud-Clone Corp., Katy, TX 77494, USA). After PBS cleansing, the DAB work solution was amplified until the color changing visualized. Digital images were photographed using an Axioplan 2 Imaging microscope (Carl Zeiss, Oberkochen, Germany).

### 2.9. Gut Microbiome Analysis by Real-Time PCR

The fecal suspensions obtained on the 21st day of the experiment were pretreated with PREP-L reagent (DNA-Technology, Moscow, Russia), which was necessary for the effective destruction of Gram-positive bacterial cell walls that dominate the microbiota and for obtaining valid research results [49]. Subsequently, DNA was isolated using the PREP-NA-PLUS kit (DNA-Technology, Moscow, Russia), and real-time PCR was performed using the Enteroflor^®^ Kiddy kit (DNA-Technology, Moscow, Russia). This reagent kit was designed for DNA detection of gut-associated microorganisms (phyla *Firmicutes*, *Proteobacteria*, *Bacteroidetes*, *Actinobacteria*, *Fusobacteria*, *Verrucomicrobia*, *Euryarchaeota*), including fungi of the genus *Candida*, by real-time PCR in DNA preparations obtained from fecal samples, in order to assess the colon microbiota composition. The obtained results were processed using RealTime_PCR 7.10 software (DNA-Technology, Moscow, Russia). It was demonstrated that the rat gut microbiome was represented by the same dominant bacterial phyla (*Firmicutes* and *Bacteroidetes*) as in humans [50]; therefore, the approach we used for the analysis was correct due to the taxonomic structure similarity.

### 2.10. Statistics

Statistical processing of the obtained results was performed in Statistica 8.0 and GraphPad Prism 8.0. The indicator distribution was determined using the Kolmogorov–Smirnov criterion. Since the data were not normally distributed, the reliability of differences between the indicators was determined using the nonparametric Mann–Whitney (differences between animals with different tolerance to hypoxia), Kruskal–Wallis and Dunn criteria (differences between animals with different tolerance to hypoxia, taking into account the dynamics of changes). The data were expressed as the median (Me) and interquartile range (25–75%). Differences were considered statistically significant at *p* < 0.05.

## 3. Results

### 3.1. Morphological and Morphometric Study of the Distal Colon

**Figure 2:**
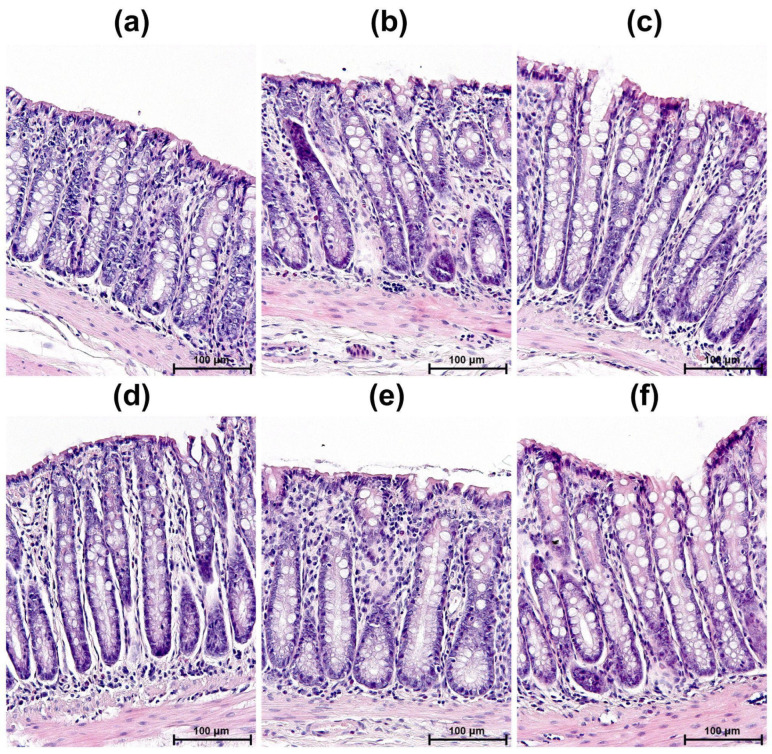
Morphological study of the distal colon in tolerant (**a**–**c**) and susceptible-to-hypoxia (**d**–**f**) Wistar rats of the control groups (**a**,**d**) and after hypoxic load at “altitudes” of 5000 m (**b**,**e**) and 7000 m (**c**,**f**) for 1 h daily for 21 days. Hematoxylin and eosin staining.

In the morphological study of colon histological sections in tolerant and susceptible-to-hypoxia animals of the control groups, no pathological changes were revealed (Figure 2a,d). The epithelial lining of the mucosa was preserved along its entire length; the crypts were deep, their lumens were narrow, and they contained many small oval goblet cells. In the lamina propria, diffusely scattered fibroblasts, fibrocytes, lymphocytes, single plasmocytes, histiocytes, and neutrophils were detected. After a hypoxic load of varying severity, no pathological changes were revealed in tolerant and susceptible-to-hypoxia Wistar rats (Figure 2b,c,e,f).

**Figure 3:**
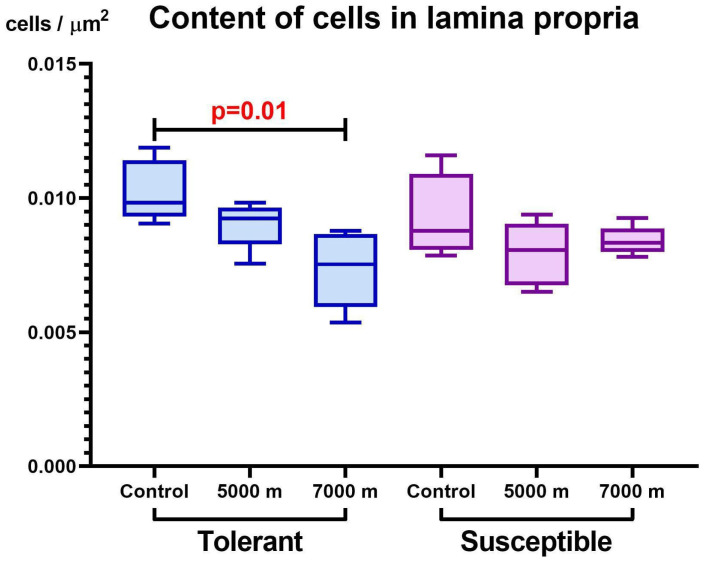
Cell content in the lamina propria in tolerant and susceptible-to-hypoxia Wistar rats of the control groups and after hypoxic load at “altitudes” of 5000 m and 7000 m for 1 h daily for 21 days. Me (25–75%). *p*–statistical significance of differences, Mann–Whitney, Kruskal–Wallis and Dunn tests.

In the morphometric study of the cell content in the lamina propria, no statistically significant differences between animals with different tolerance to hypoxia were revealed. At the same time, only in rats tolerant to hypoxia, a decrease in the number of cells in the lamina propria was revealed after a hypoxic load at an “altitude” of 7000 m for 1 h daily for 21 days in comparison with the control group (Figure 3).

### 3.2. Histochemical Study of Goblet Cells

**Figure 4:**
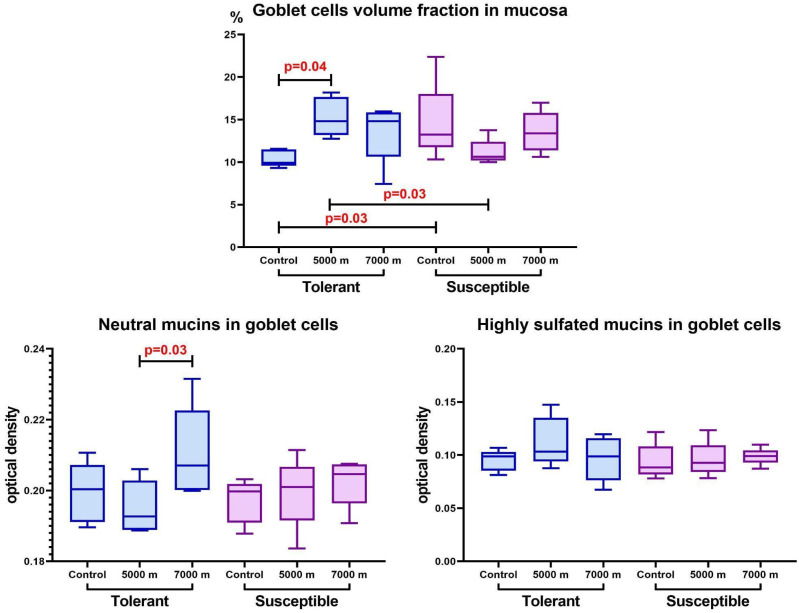
Goblet cell volume fraction and the content of neutral and highly sulfated mucins in them in tolerant and susceptible-to-hypoxia Wistar rats of the control groups and after hypoxic load at “altitudes” of 5000 m and 7000 m for 1 h daily for 21 days. Me (25–75%). *p*–statistical significance of differences, Mann–Whitney, Kruskal–Wallis and Dunn tests.

When analyzing histological specimens of the distal colon stained with PAS, susceptible-to-hypoxia animals of the control group demonstrated a greater volume fraction of goblet cells in comparison with tolerant-to-hypoxia animals (Figure 4). At the same time, in tolerant rats to hypoxia, after a hypoxic load at an “altitude” of 5000 m for 1 h daily for 21 days, an increase in the goblet cell volume fraction was revealed in comparison with the control group, and the indicators were higher in comparison with susceptible-to-hypoxia rats subjected to the same hypoxic load. No changes in the goblet cell volume fraction in comparison with the control group were revealed after hypoxic exposure in susceptible-to-hypoxia animals.

**Figure 5:**
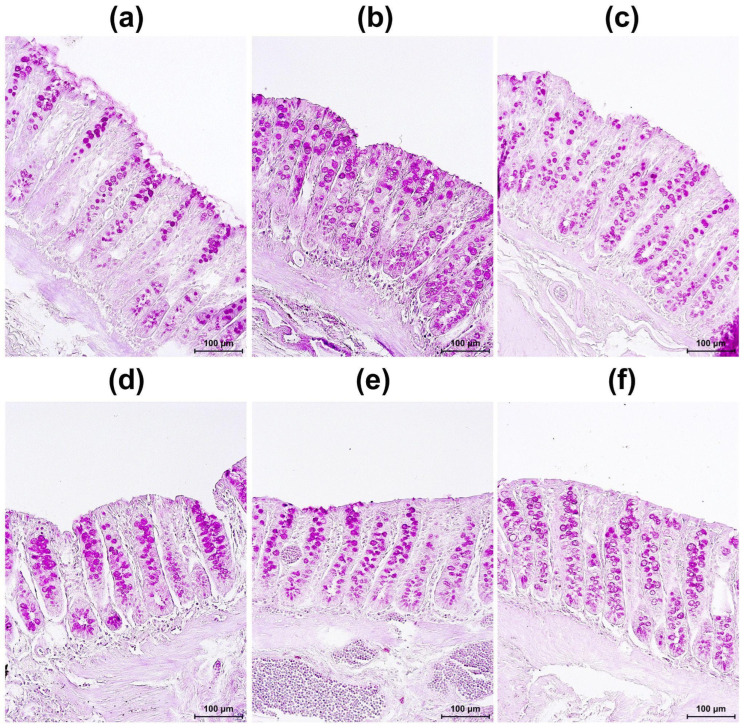
Goblet cells containing neutral mucins in the distal colon in tolerant (**a**–**c**) and susceptible-to-hypoxia (**d**–**f**) Wistar rats of the control groups (**a**,**d**) and after hypoxic load at “altitudes” of 5000 m (**b**,**e**) and 7000 m (**c**,**f**) for 1 h daily for 21 days. PAS reaction.

**Figure 6:**
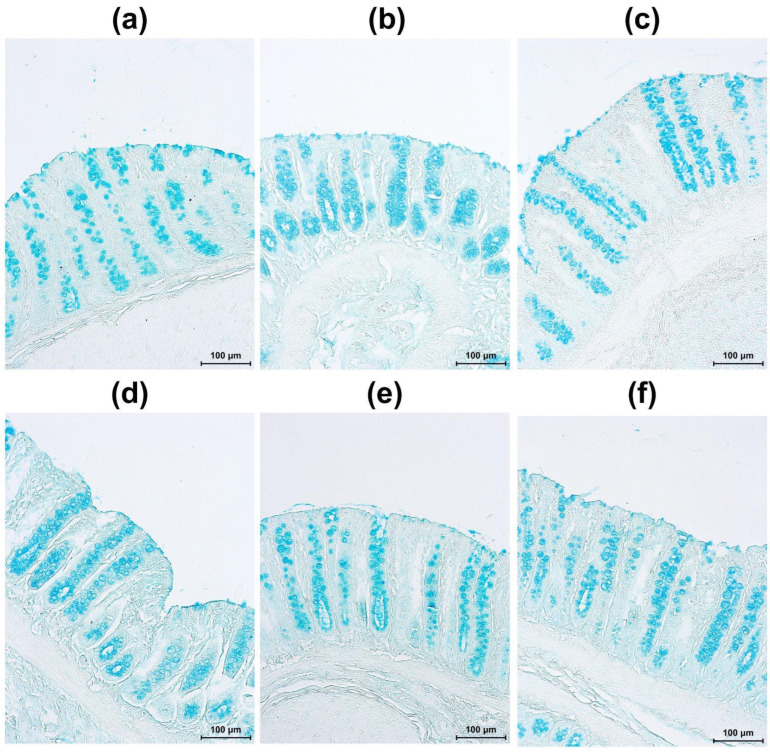
Goblet cells containing highly sulfated mucins in the distal colon in tolerant (**a**–**c**) and susceptible-to-hypoxia (**d**–**f**) Wistar rats of the control groups (**a**,**d**) and after hypoxic load at “altitudes” of 5000 m (**b**,**e**) and 7000 m (**c**,**f**) for 1 h daily for 21 days. Alcian blue staining.

Furthermore, only in rats tolerant to hypoxia, an increase in the content of neutral mucins in goblet cells was revealed (Figure 5 and Figure 6) under load at an “altitude” of 7000 m for 1 h daily for 21 days in comparison with the “altitude” of 5000 m, while the content of highly sulfated mucins did not change (Figure 5 and Figure 6).

### 3.3. Immunohistochemistry

**Figure 7:**
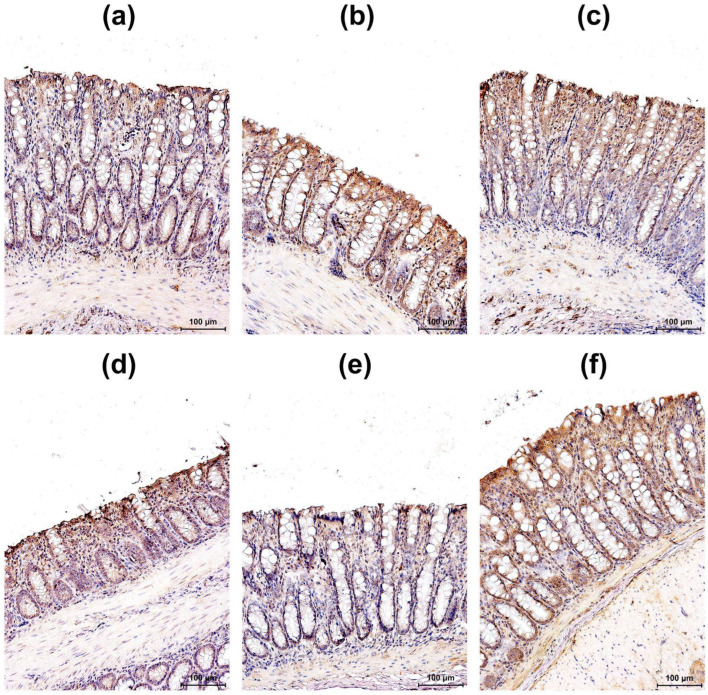
Immunohistochemical detection of HIF-1α protein in the distal colon in tolerant (**a**–**c**) and susceptible-to-hypoxia (**d**–**f**) Wistar rats of the control groups (**a**,**d**) and after hypoxic load at “altitudes” of 5000 m (**b**,**e**) and 7000 m (**c**,**f**) for 1 h daily for 21 days. (**a**,**d**) Control groups, the reaction was weakly expressed; (**b**) tolerant to hypoxia after hypoxic load at the “altitude” of 5000 m, the reaction was more pronounced than in the control group and in susceptible to hypoxia; (**e**) susceptible to hypoxia after hypoxic load at the “altitude” of 5000 m, the reaction was weakly expressed; (**c**) tolerant to hypoxia after hypoxic load at the “altitude” of 7000 m, the reaction was more pronounced than in the control group; (**f**) susceptible to hypoxia after hypoxic load at the “altitude” of 7000 m, the reaction was more pronounced than in the control group, at the “altitude” of 5000 m, and in tolerant to hypoxia under the same hypoxic conditions. Immunohistochemical reaction with antibodies to HIF-1α, counterstained with hematoxylin.

When detecting the HIF-1α protein in the distal colon, a more pronounced reaction was detected in susceptible-to-hypoxia rats after hypoxic exposure at an “altitude” of 7000 m for 1 h daily for 21 days in comparison with the control group and the “altitude” of 5000 m (Figure 7). Furthermore, at the “altitude” of 5000 m, a more pronounced reaction was revealed in tolerant-to-hypoxia animals in comparison with susceptible animals, while at the “altitude” of 7000 m, the opposite was observed.

**Figure 8:**
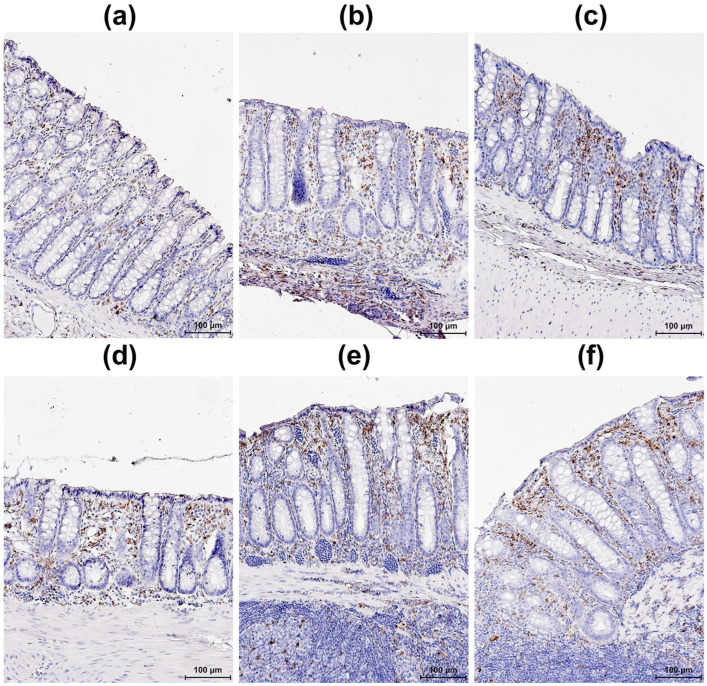
Immunohistochemical detection of CD68+ cells in the distal colon in tolerant (**a**–**c**) and susceptible-to-hypoxia (**d**–**f**) Wistar rats of the control groups (**a**,**d**) and after hypoxic load at “altitudes” of 5000 m (**b**,**e**) and 7000 m (**c**,**f**) for 1 h daily for 21 days. Immunohistochemical reaction with antibodies to CD68+, counterstained with hematoxylin.

To assess the cellular composition of the lamina propria, we stained sections of the distal colon with antibodies to CD68+ to detect macrophages (Figure 8).

### 3.4. Gut Microbiome Analysis

**Figure 9:**
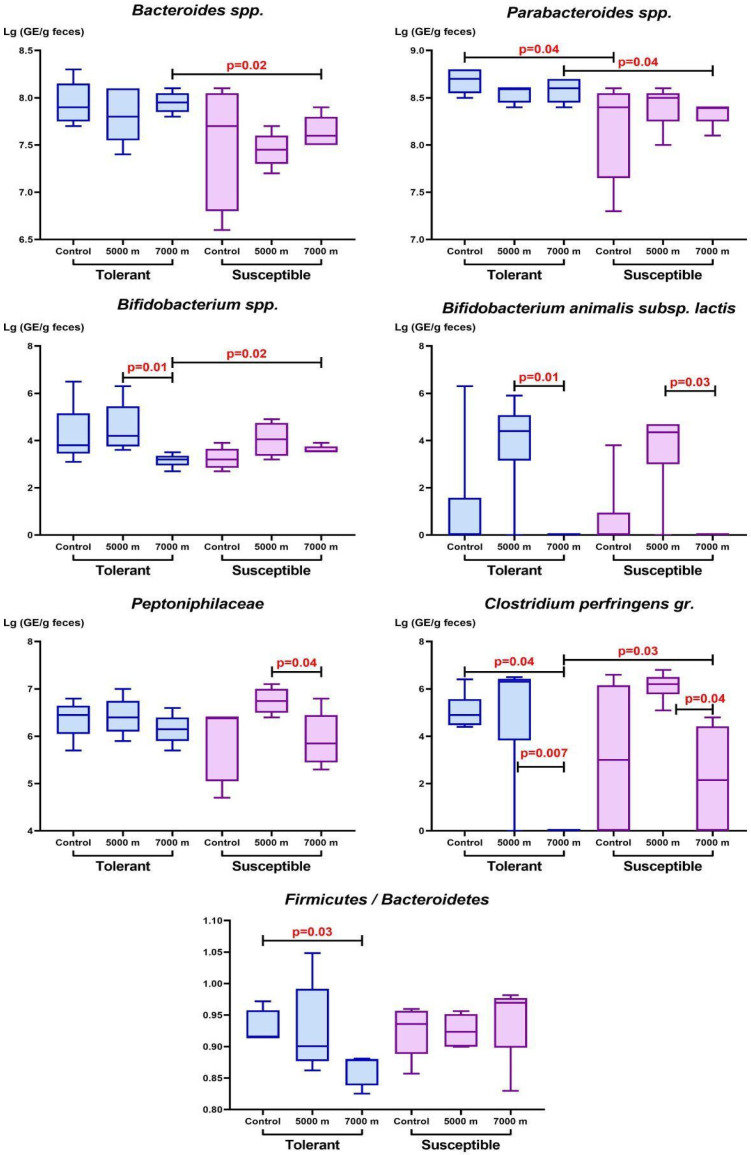
Abundance of *Bacteroides* spp., *Bifidobacterium animalis subsp. lactis*, *Bifidobacterium* spp., *Parabacteroides* spp., *Peptoniphilaceae*, *Clostridium perfringens gr.*, and the *Firmicutes*/*Bacteroidetes* ratio in the feces of tolerant and susceptible-to-hypoxia Wistar rats of the control groups and after hypoxic load at “altitudes” of 5000 m and 7000 m for 1 h daily for 21 days. Me (25–75%). p–statistical significance of differences, Mann–Whitney, Kruskal–Wallis and Dunn tests.

According to the results of the gut microbiome study, out of the 35 bacterial taxa investigated (Table S1), statistically significant differences were revealed only for the abundance of *Bacteroides* spp., *Bifidobacterium animalis subsp. lactis*, *Bifidobacterium* spp., *Parabacteroides* spp., *Peptoniphilaceae*, and *Clostridium perfringens gr.* (Figure 9). Tolerant-to-hypoxia rats of the control group, in comparison with susceptible rats, were characterized by a higher abundance of *Parabacteroides* spp. After a hypoxic load at an “altitude” of 5000 m for 1 h daily for 21 days, no statistically significant changes in the microbiome composition were revealed, regardless of the tolerance to hypoxia. At the same time, after load at an “altitude” of 7000 m, in comparison with the control, in tolerant-to-hypoxia rats, a decrease in the abundance of *Clostridium perfringens gr.* and the *Firmicutes*/*Bacteroidetes* ratio was revealed, while no changes were detected in susceptible-to-hypoxia rats. After load at an “altitude” of 7000 m, in tolerant-to-hypoxia rats, in comparison with susceptible-to-hypoxia rats, the abundance of *Bacteroides* spp. and *Parabacteroides* spp. was higher, but the abundance of *Bifidobacterium* spp. and *Clostridium perfringens gr.* was lower. After load at an “altitude” of 7000 m, in comparison with the “altitude” of 5000 m, regardless of the tolerance to oxygen deficiency, a decrease in *Bifidobacterium animalis subsp. Lactis* and *Clostridium perfringens gr.* were revealed. At the same time, only in rats tolerant to hypoxia, a decrease in the abundance of *Bifidobacterium* spp. was noted, and in susceptible-to-hypoxia rats—*Peptoniphilaceae*.

**Figure 10:**
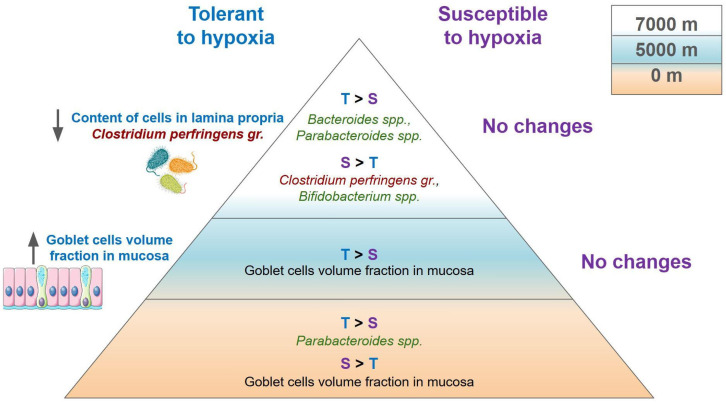
Summarized data of the study of the colon histophysiological features and the gut microbiome in tolerant and susceptible to oxygen deficiency Wistar rats after prolonged intermittent hypoxic exposure. Green color—representatives of the normal microbiota, red color—representatives of the opportunistic microbiota, up arrow—increase in the indicator relative to the control group, down arrow—decrease in the indicator relative to the control group.

The summarized data are presented in Figure 10.

## 4. Discussion

In our study, data on the features of the intestinal barrier and microbiome in animals with different tolerance to oxygen deficiency, which were not subjected to a sublethal hypoxic load in a decompression chamber, were obtained for the first time.

Animals susceptible to hypoxia of the control group, in comparison with the tolerant, were characterized by a greater volume fraction of goblet cells and a low *Parabacteroides* spp. abundance. *Parabacteroides* spp. is a genus of Gram-negative, obligate anaerobic bacteria that are an important part of the normal intestinal microflora of mammals [51]. Representatives of this genus synthesized antimicrobial substances, such as apigenin, which prevented intestinal colonization by pathogenic bacteria [52]. Furthermore, *Parabacteroides* spp. participated in the conversion of bile and higher fatty acids, which modulated lipid and glucose metabolism, preventing insulin resistance and obesity [53]. Pan M. et al. (2022) demonstrated that two *Parabacteroides* strains (*P. johnsonii* and *P. distasonis*) could degrade mucin [54]. On the Caco-2 cell line, it was demonstrated that these two strains, when co-incubated for 12 h, also modulated the barrier function of epithelial tight junctions—they increased Transepithelial Electrical Resistance (TEER) by approximately 10%. Furthermore, when incubating Caco-2 with the pro-inflammatory cytokine IL-1β (10 ng/mL), TEER decreased to 81.7% after 12 h and to 77.2% after 24 h in comparison with the control (100%), and with the addition of *P. distasonis*, this indicator not only did not decrease but, on the contrary, increased to ~106%, which indicated the protective function of these bacteria. The greater volume fraction of goblet cells, the main producers of mucins and intestinal mucus, in susceptible-to-hypoxia rats, was probably aimed at strengthening the mechanical protection of the intestinal barrier against the background of a low *Parabacteroides* spp. abundance.

In our study, after a hypoxic load at an “altitude” of 5000 m for 1 h daily for 21 days, the goblet cell volume fraction was lower in susceptible-to-hypoxia rats in comparison with tolerant rats, in which it increased in comparison with the control group. At the same time, no statistically significant changes in the gut microbiome composition were revealed. According to data [55] in an experiment on female C57BL/6 mice subjected to hypobaric hypoxia at an “altitude” of 5000 m for 7 days, it was demonstrated that exposure to oxygen deficiency led to minor changes in the colon structure, including a decrease in the number of goblet cells and mucin content per crypt. In comparison with the control group, a higher content of diamine oxidase and D-lactate was revealed in the blood serum, an increased level of which correlated with intestinal mucosal barrier dysfunction and was an indicator of changes in its permeability. Furthermore, in animals subjected to hypoxia, the expression levels of the pro-inflammatory cytokines *Il1b* and *Il6* in intestinal tissues were higher. The authors demonstrated that in response to hypoxic exposure, the Notch signaling pathway was activated, which was accompanied by an increase in the transcriptional repressor Hes-1 content and a decrease in the Math-1 factor [55], which could promote intestinal stem cell differentiation into absorptive colonocytes and led to a decrease in the goblet cell population; however, the exact molecular mechanisms have not yet been established. In another experiment on male C57BL/6 mice subjected to hypobaric hypoxia at an “altitude” of 6000 m for 7 days, it was also demonstrated that after exposure, the number of goblet cells in the colon decreased by 27.6% [56]. Next, the authors conducted a transcriptomic study of goblet cells, according to which the expression levels of the main markers of these cells, *Fcgbp* (Fcγ-binding protein) and *Clca1* (calcium-activated chloride channel regulator 1), decreased after hypobaric hypoxia exposure, while *Hif1a* increased. In an experiment on male Sprague–Dawley rats subjected to hypoxia at an “altitude” of 7000 m for 3 days, it was demonstrated that after exposure to oxygen deficiency, the crypt depth and the relative density of goblet cells in the colon decreased, which indicated mucus layer damage [57]. At the same time, the abundance of the *Prevotella_NK3B31_group* and *Ruminococcus* species decreased significantly in comparison with the control group, which the authors interpreted as a manifestation of dysbiosis. Thus, in the studies characterized above, in response to hypoxic exposure, changes were revealed in the gut that indicated barrier damage—a decrease in the number of goblet cells and mucins, an increase in diamine oxidase and D-lactate content in the blood serum, and the expression levels of the pro-inflammatory cytokines *Il1b* and *Il6* in intestinal tissues. However, in our experiment, in tolerant-to-hypoxia rats, an increase in the number of goblet cells was revealed, which could be associated with barrier function strengthening. Probably, the revealed contradiction was associated with the selected hypoxic load model—in our work, it represented a prolonged intermittent exposure, while in studies [55,56]—a chronic one. It is known that cellular responses to acute and chronic hypoxia differ [58]; therefore, the exposure severity and duration influenced the experimental results. Furthermore, ref. [55] performed the experiment on females, and [56,57]—on males, and did not take into account the initial animal tolerance to oxygen deficiency, which could also have affected the experiment results.

After load at an “altitude” of 7000 m, in tolerant-to-hypoxia rats, in comparison with the control group, a decrease in the number of cells in the lamina propria, the abundance of *Clostridium perfringens gr.*, and the *Firmicutes/Bacteroidetes* ratio was revealed, while no changes were detected in susceptible animals. At the same time, in tolerant rats to hypoxia, in comparison with susceptible rats, the abundance of *Bacteroides* spp. and *Parabacteroides* spp. was higher, but the abundance of *Bifidobacterium* spp. and *Clostridium perfringens gr.* was lower. *Clostridium perfringens gr.* represented a group of bacteria whose 16S rRNA sequence was close to *Clostridium perfringens*. Furthermore, they produced substances with neurotoxic, hemolytic, and enterotoxigenic activity [59], and an increase in their number could lead to the development of acute enteritis and necrotizing enterocolitis [60]. The low abundance of this taxon’s representatives in animals tolerant to hypoxia was a favorable sign that reduced the risk of damage to the intestinal barrier. At the same time, in susceptible-to-hypoxia rats, the high abundance of *Clostridium perfringens gr.* against the background of a low abundance of *Parabacteroides* spp. could be the cause of the intestinal barrier function weakening [52,60], and could also condition a more severe course of acute and chronic ulcerative colitis and colitis-associated colorectal cancer [24,25,26]. At the same time, the low abundance of *Bifidobacterium* spp., commensal bacteria maintaining intestinal homeostasis and possessing immunomodulatory properties [61], in tolerant-to-hypoxia rats after load at an “altitude” of 7000 m was combined with a high abundance of *Bacteroides* spp. and *Parabacteroides* spp., which probably compensated for the key metabolic and protective functions and maintained intestinal homeostasis under hypoxic stress conditions [62]. The main common function of bacteria belonging to these genera was the support of the intestinal barrier function. *Bifidobacterium* spp. enhanced tight junction protein synthesis, induced regulatory T-lymphocytes, and suppressed pro-inflammatory Th2- and Th17-induced responses [61]. At the same time, *Bacteroides* spp. and *Parabacteroides* spp. also possessed immunomodulatory properties. Thus, *P. distasonis* reduced the severity of the inflammatory response through succinate and secondary bile acid formation, which activated intestinal gluconeogenesis and the farnesoid X receptor (FXR) signaling pathway, contributing to hyperglycemia reduction and the restoration of the intestinal barrier integrity [53]. At the same time, *Bacteroides* spp. produced short-chain fatty acids, which, through the G-protein-coupled receptor GPR43, induced regulatory T-lymphocyte differentiation and the anti-inflammatory cytokine IL-10 production [63]. Furthermore, in tolerant animals to hypoxia, the decrease in the number of cells in the lamina propria could be a consequence of the response to prolonged intermittent hypoxic exposure aimed at strengthening the epithelial barrier, leading to a decrease in luminal antigen translocation. It was demonstrated that HIF protein stabilization under hypoxic conditions promoted antimicrobial peptide production and the maintenance of intercellular junction integrity, which prevented the penetration of bacteria and their components [20].

After load for 1 h daily for 21 days at an “altitude” of 7000 m in comparison with the “altitude” of 5000 m, regardless of the tolerance to oxygen deficiency, a decrease in *Bifidobacterium animalis subsp. lactis* and *Clostridium perfringens gr.* were revealed. At the same time, only in rats tolerant to hypoxia, an increase in the content of neutral mucins in goblet cells and a decrease in the abundance of *Bifidobacterium* spp. were noted, and in susceptible-to-hypoxia rats—*Peptoniphilaceae*. In rats tolerant to hypoxia, against the background of a high abundance of *Bacteroides* spp. and *Parabacteroides* spp., the decrease in the abundance of *Bifidobacterium* spp. and the number of cells in the lamina propria could reflect an adaptive rearrangement of immune homeostasis aimed at reducing the severity of the inflammatory response. At the same time, in susceptible-to-hypoxia animals, a decrease in the opportunistic bacteria of the *Peptoniphilaceae* family was revealed against the background of a high abundance of *Clostridium perfringens gr.* and the absence of pathological changes in the distal colon. This could indicate that the microbiome change in these rats was not accompanied by an adequate immune reaction.

This study had a number of limitations. To assess the state of the epithelial barrier of the distal colon, we used histological, histochemical, and immunohistochemical methods; however, we did not analyze the expression levels and protein content of tight junction proteins (claudins, occludins) and mucins, which were the main components of the intestinal mucus. It is also necessary to evaluate the cellular composition of the lamina propria by immunohistochemistry and quantitative assessment of immune cell subpopulations. Furthermore, for the microbiome study, we used the PCR method, which, unlike metagenomic sequencing, did not allow the assessment of complete taxonomic diversity. However, we managed to determine not only the relative but also the absolute abundance of clinically significant microorganisms, which allowed large taxon identification and the detection of several key microbiota representatives, obtaining unambiguously interpretable results using a routine approach and without complex bioinformatic processing. Furthermore, we investigated the influence of two hypoxic exposure regimes and, thus, evaluated the dose-dependence of the revealed effects. Additional research should be conducted to identify the molecular–biological mechanisms regulating the cellular response to oxygen deficiency along the gut–microbiome axis in organisms with different tolerance to hypoxia. Analysis of the gut microbiota has the potential to identify specific microbial species whose abundance correlates with individual tolerance to oxygen deficiency. In particular, modulating the levels of opportunistic pathogens, which are found at higher relative abundance in susceptible-to-hypoxia organisms, could enhance their tolerance to oxygen deficiency. Such interventions may reduce both the risk and the severity of acute mountain sickness, opening new opportunities for predictive diagnostics and targeted microbiota-based prevention in high-altitude medicine.

## 5. Conclusions

For the first time, we performed an analysis of the intestinal epithelial barrier histophysiological features and the microbiome in rats with different tolerance to oxygen deficiency. In comparison with tolerant-to-hypoxia rats, susceptible animals demonstrated a greater volume fraction of goblet cells and a low abundance of *Parabacteroides* spp. under normoxic conditions. Intermittent hypoxic exposure induced multidirectional changes depending on the initial animal tolerance to hypoxia and the regimen severity. In animals tolerant to hypoxia, an increase in the goblet cell volume fraction was detected after exposure at an “altitude” of 5000 m for 1 h daily for 21 days, while at an “altitude” of 7000 m, a decrease in the number of cells in the lamina propria and *Clostridium perfringens gr.* abundance, as well as a reduction in the *Firmicutes/Bacteroidetes* ratio, was observed. In susceptible-to-hypoxia animals, a higher abundance of *Clostridium perfringens gr.* in comparison with tolerant-to-hypoxia rats was revealed after exposure at an “altitude” of 7000 m, with no structural changes in the intestinal wall. Thus, intermittent hypoxic exposure led to a rearrangement of the gut microbiome and the intestinal barrier morphofunctional characteristics, and the severity of these changes depended on the initial organism tolerance to oxygen deficiency and the hypoxic regime severity. The obtained data should be taken into account when conducting research using animals with different tolerances to hypoxia.

## Figures and Tables

**Table 1:** *Hif1a*, *Epas1*, and *Hif3a* expression levels in peripheral blood leukocytes in tolerant and susceptible-to-hypoxia animals. Me (25–75%). *p*–statistical significance of differences, Kruskal–Wallis and Dunn tests.

Gene	Tolerant	Susceptible	*p*-Value
*Hif1a*	0.00059 (0.00029–0.001522)	0.03176 (0.009949–0.07838)	0.0001
*Epas1*	0.01498 (0.008382–0.02866)	0.2788 (0.1712–0.4333)	0.0001
*Hif3a*	0.002369 (0.001238–0.00706)	0.3716 (0.1708–0.5789)	0.0001

## Data Availability

The original contributions presented in this study are included in the article/Supplementary Material (Table S2). Further inquiries can be directed to the corresponding author.

## Supplementary Materials

The following supporting information can be downloaded at: https://www.mdpi.com/article/10.3390/biom16070935/s1, Table S1: Results of microbiome analysis, Table S2: Minimal dataset.

## Abbreviations

The following abbreviations are used in this manuscript:

HIF	Hypoxia-Inducible Factor
PAS	Periodic Acid-Schiff
TEER	Transepithelial Electrical Resistance

## References

[1] Yu Y., Zhang Y., Yang Y. (2026). From mechanisms to therapeutics: Molecular insights into gastrointestinal injury under high-altitude hypoxia. Front. Microbiol..

[2] Wang Y., Shi Y., Li W., Wang S., Zheng J., Xu G., Li G., Shen X., Yang J. (2022). Gut microbiota imbalance mediates intestinal barrier damage in high-altitude exposed mice. FEBS J..

[3] Kleessen B., Schroedl W., Stueck M., Richter A., Rieck O., Krueger M. (2005). Microbial and immunological responses relative to high-altitude exposure in mountaineers. Med. Sci. Sports Exerc..

[4] Qi P., Lv J., Bai L.-H., Yan X.-D., Zhang L. (2023). Effects of Hypoxemia by Acute High-Altitude Exposure on Human Intestinal Flora and Metabolism. Microorganisms.

[5] Rodríguez F.A., Ventura J.L., Casas M., Casas H., Pagés T., Rama R., Ricart A., Palacios L., Viscor G. (2000). Erythropoietin acute reaction and haematological adaptations to short, intermittent hypobaric hypoxia. Eur. J. Appl. Physiol..

[6] Hamer H.M., Jonkers D., Venema K., Vanhoutvin S., Troost F.J., Brummer R.J. (2008). Review article: The role of butyrate on colonic function. Aliment. Pharmacol. Ther..

[7] Blouin J.-M., Penot G., Collinet M., Nacfer M., Forest C., Laurent-Puig P., Coumoul X., Barouki R., Benelli C., Bortoli S. (2011). Butyrate elicits a metabolic switch in human colon cancer cells by targeting the pyruvate dehydrogenase complex. Int. J. Cancer.

[8] Kelly C.J., Zheng L., Campbell E.L., Saeedi B., Scholz C.C., Bayless A.J., Wilson K.E., Glover L.E., Kominsky D.J., Magnuson A. (2015). Crosstalk between Microbiota-Derived Short-Chain Fatty Acids and Intestinal Epithelial HIF Augments Tissue Barrier Function. Cell Host Microbe.

[9] Rivera-Chávez F., Zhang L.F., Faber F., Lopez C.A., Byndloss M.X., Olsan E.E., Xu G., Velazquez E.M., Lebrilla C.B., Winter S.E. (2016). Depletion of Butyrate-Producing Clostridia from the Gut Microbiota Drives an Aerobic Luminal Expansion of Salmonella. Cell Host Microbe.

[10] Espey M.G. (2013). Role of oxygen gradients in shaping redox relationships between the human intestine and its microbiota. Free Radic. Biol. Med..

[11] Karhausen J., Furuta G.T., Tomaszewski J.E., Johnson R.S., Colgan S.P., Haase V.H. (2004). Epithelial hypoxia-inducible factor-1 is protective in murine experimental colitis. J. Clin. Investig..

[12] Cummins E.P., Seeballuck F., Keely S.J., Mangan N.E., Callanan J.J., Fallon P.G., Taylor C.T. (2008). The hydroxylase inhibitor dimethyloxalylglycine is protective in a murine model of colitis. Gastroenterology.

[13] Bandarra D., Biddlestone J., Mudie S., Müller H.-A.J., Rocha S. (2015). HIF-1α restricts NF-κB-dependent gene expression to control innate immunity signals. Dis. Model. Mech..

[14] Van Itallie C.M., Anderson J.M. (2014). Architecture of tight junctions and principles of molecular composition. Semin. Cell Dev. Biol..

[15] Muenchau S., Deutsch R., de Castro I.J., Hielscher T., Heber N., Niesler B., Lusic M., Stanifer M.L., Boulant S. (2019). Hypoxic Environment Promotes Barrier Formation in Human Intestinal Epithelial Cells through Regulation of MicroRNA 320a Expression. Mol. Cell. Biol..

[16] Johansson M.E.V., Phillipson M., Petersson J., Velcich A., Holm L., Hansson G.C. (2008). The inner of the two Muc2 mucin-dependent mucus layers in colon is devoid of bacteria. Proc. Natl. Acad. Sci. USA.

[17] Holmén Larsson J.M., Thomsson K.A., Rodríguez-Piñeiro A.M., Karlsson H., Hansson G.C. (2013). Studies of mucus in mouse stomach, small intestine, and colon. III. Gastrointestinal Muc5ac and Muc2 mucin O-glycan patterns reveal a regiospecific distribution. Am. J. Physiol. Gastrointest. Liver Physiol..

[18] Louis N.A., Hamilton K.E., Canny G., Shekels L.L., Ho S.B., Colgan S.P. (2006). Selective induction of mucin-3 by hypoxia in intestinal epithelia. J. Cell. Biochem..

[19] Dilly A.K., Lee Y.J., Zeh H.J., Guo Z.S., Bartlett D.L., Choudry H.A. (2016). Targeting hypoxia-mediated mucin 2 production as a therapeutic strategy for mucinous tumors. Transl. Res..

[20] Glover L.E., Lee J.S., Colgan S.P. (2016). Oxygen metabolism and barrier regulation in the intestinal mucosa. J. Clin. Investig..

[21] Adak A., Maity C., Ghosh K., Mondal K.C. (2014). Alteration of predominant gastrointestinal flora and oxidative damage of large intestine under simulated hypobaric hypoxia. Z. Gastroenterol..

[22] Han Y., Xu J., Yan Y., Zhao X. (2022). Dynamics of the gut microbiota in rats after hypobaric hypoxia exposure. PeerJ.

[23] Dzhalilova D.S., Polyakova M.A., Diatroptov M.E., Zolotova N.A., Makarova O.V. (2018). Morphological changes in the colon and composition of peripheral blood lymphocytes in acute colitis in mice with different resistance to hypoxia. Mol. Med..

[24] Dzhalilova D.S., Zolotova N.A., Polyakova M.A., Diatroptov M.E., Dobrynina M.T., Makarova O.V. (2018). Morphological features of the inflammatory process and subpopulation pattern of peripheral blood lymphocytes during chronic colitis in mice exhibiting different responses to hypoxia. Clin. Exp. Morphol..

[25] Dzhalilova D., Silina M., Tsvetkov I., Kosyreva A., Zolotova N., Gantsova E., Kirillov V., Fokichev N., Makarova O. (2024). Changes in the Expression of Genes Regulating the Response to Hypoxia, Inflammation, Cell Cycle, Apoptosis, and Epithelial Barrier Functioning during Colitis-Associated Colorectal Cancer Depend on Individual Hypoxia Tolerance. Int. J. Mol. Sci..

[26] Dzhalilova D., Silina M., Kosyreva A., Fokichev N., Makarova O. (2025). Morphofunctional changes in the immune system in colitis-associated colorectal cancer in tolerant and susceptible to hypoxia mice. PeerJ.

[27] Richalet J.-P., Lhuissier F.J. (2015). Aging, tolerance to high altitude, and cardiorespiratory response to hypoxia. High Alt. Med. Biol..

[28] Jia N., Chen C., Chen Q., Liu J., Shen Z., Liu Y., Pei C., Wang Y., Huang D., Wang F. (2025). Acute high-altitude illness: Risk factors, susceptibility prediction, and personalized prevention and treatment. Front. Med..

[29] Dzhalilova D., Makarova O. (2020). Differences in Tolerance to Hypoxia: Physiological, Biochemical, and Molecular-Biological Characteristics. Biomedicines.

[30] Bezrukov V.V., Paramononva G.I., Rushkevich S.N., Timchenko A.N., Utko N.A., Kholin V.A. (2012). Some physiological indices and life expectancy in rats with different resistance to hypoxia. Probl. Stareniya Dolgoletiya.

[31] Sanotskaya N.V., Matsievskii D.D., Lebedeva M.A. (2004). Changes in hemodynamics and respiration in rats with different resistance to acute hypoxia. Bull. Exp. Biol. Med..

[32] Pavlik L.L., Mikheeva I.B., Al’-Mugkhrabi Y.M., Berest V.P., Kirova Y.I., Germanova E.L., Luk’yanova L.D., Mironova G.D. (2018). Specific Features of Immediate Ultrastructural Changes in Brain Cortex Mitochondria of Rats with Different Tolerance to Hypoxia under Various Modes of Hypoxic Exposures. Bull. Exp. Biol. Med..

[33] Mironova G.D., Pavlik L.L., Kirova Y.I., Belosludtseva N.V., Mosentsov A.A., Khmil N.V., Germanova E.L., Lukyanova L.D. (2019). Effect of hypoxia on mitochondrial enzymes and ultrastructure in the brain cortex of rats with different tolerance to oxygen shortage. J. Bioenerg. Biomembr..

[34] Jain K., Suryakumar G., Prasad R., Ganju L. (2013). Upregulation of cytoprotective defense mechanisms and hypoxia-responsive proteins imparts tolerance to acute hypobaric hypoxia. High Alt. Med. Biol..

[35] Padhy G., Sethy N.K., Ganju L., Bhargava K. (2013). Abundance of plasma antioxidant proteins confers tolerance to acute hypobaric hypoxia exposure. High Alt. Med. Biol..

[36] Zolotova N.A., Dzhalilova D.S., Khochanskiy D.N., Tsvetkov I.S., Kosyreva A.M., Ponomarenko E.A., Diatroptova M.A., Mikhailova L.P., Mkhitarov V.A., Makarova O.V. (2021). Morphofunctional Changes in Colon after Cold Stress in Male C57BL/6 Mice Susceptible and Tolerant to Hypoxia. Bull. Exp. Biol. Med..

[37] Kirova Y.I., Germanova E.L., Lukyanova L.D. (2013). Phenotypic features of the dynamics of HIF-1α levels in rat neocortex in different hypoxia regimens. Bull. Exp. Biol. Med..

[38] Dzhalilova D.S., Kosyreva A.M., Diatroptov M.E., Ponomarenko E.A., Tsvetkov I.S., Zolotova N.A., Mkhitarov V.A., Khochanskiy D.N., Makarova O.V. (2019). Dependence of the severity of the systemic inflammatory response on resistance to hypoxia in male Wistar rats. J. Inflamm. Res..

[39] Makarova O.V., Mikhailova L.P., Sladkopevtzev A.S., Zykova I.E., Turusina T.A. (1992). Influence of normo-barometric hypoxia on the cell content of broncho-alveolar lavage and phagocytic capacity of neutrophils and macrophages from Wistar rats’lungs. Pulmonologiya.

[40] Kirillova M., Dzhalilova D., Maiak M., Kirillov V., Tsvetkov I., Fokichev N., Makarova O. (2026). MicroRNAs and genes regulating responses to hypoxia and inflammation expression levels in blood leukocytes as potential biomarkers of initial oxygen deficiency tolerance. Front. Mol. Biosci..

[41] Kosyreva A.M., Dzhalilova D.S., Makarova O.V., Tsvetkov I.S., Zolotova N.A., Diatroptova M.A., Ponomarenko E.A., Mkhitarov V.A., Khochanskiy D.N., Mikhailova L.P. (2020). Sex differences of inflammatory and immune response in pups of Wistar rats with SIRS. Sci. Rep..

[42] Dzhalilova D.S., Kosyreva A.M., Tsvetkov I.S., Zolotova N.A., Mkhitarov V.A., Mikhailova L.P., Makarova O.V. (2020). Morphological and Functional Peculiarities of the Immune System of Male and Female Rats with Different Hypoxic Resistance. Bull. Exp. Biol. Med..

[43] Dzhalilova D., Kosyreva A., Vishnyakova P., Zolotova N., Tsvetkov I., Mkhitarov V., Mikhailova L., Kakturskiy L., Makarova O. (2021). Age-related differences in hypoxia-associated genes and cytokine profile in male Wistar rats. Heliyon.

[44] Livak K.J., Schmittgen T.D. (2001). Analysis of relative gene expression data using real-time quantitative PCR and the 2(-Delta Delta C(T)) Method. Methods.

[45] Meerson F.Z. (1981). Adaptation, Stress and Prevention.

[46] Barnes L.A., Mesarwi O.A., Sanchez-Azofra A. (2022). The Cardiovascular and Metabolic Effects of Chronic Hypoxia in Animal Models: A Mini-Review. Front. Physiol..

[47] Girard O., Duan R., Suzuki K., Yan X. (2022). Editorial: Hypoxia and exercise: Tissue specific and systemic adaptive responses. Front. Physiol..

[48] Bankhead P., Loughrey M.B., Fernández J.A., Dombrowski Y., McArt D.G., Dunne P.D., McQuaid S., Gray R.T., Murray L.J., Coleman H.G. (2017). QuPath: Open source software for digital pathology image analysis. Sci. Rep..

[49] Han Z., Sun J., Lv A., Wang A. (2019). Biases from different DNA extraction methods in intestine microbiome research based on 16S rDNA sequencing: A case in the koi carp, *Cyprinus carpio* var. *Koi*. MicrobiologyOpen.

[50] Hillman E.T., Lu H., Yao T., Nakatsu C.H. (2017). Microbial Ecology along the Gastrointestinal Tract. Microbes Environ..

[51] Cui Y., Zhang L., Wang X., Yi Y., Shan Y., Liu B., Zhou Y., Lü X. (2022). Roles of intestinal Parabacteroides in human health and diseases. FEMS Microbiol. Lett..

[52] Gu P., Wei R., Liu R., Yang Q., He Y., Guan J., He W., Li J., Zhao Y., Xie L. (2025). Aging-induced Alternation in the Gut Microbiota Impairs Host Antibacterial Defense. Adv. Sci..

[53] Wang K., Liao M., Zhou N., Bao L., Ma K., Zheng Z., Wang Y., Liu C., Wang W., Wang J. (2019). Parabacteroides distasonis Alleviates Obesity and Metabolic Dysfunctions via Production of Succinate and Secondary Bile Acids. Cell Rep..

[54] Pan M., Barua N., Ip M. (2022). Mucin-degrading gut commensals isolated from healthy faecal donor suppress intestinal epithelial inflammation and regulate tight junction barrier function. Front. Immunol..

[55] Jia R., Han Y., Zhu Q., Zhang J., Zhang H., Ka M., Ma Y., Gamah M., Zhang W. (2025). Activation of notch signaling pathway is a potential mechanism for mucin2 reduction and intestinal mucosal barrier dysfunction in high-altitude hypoxia. Sci. Rep..

[56] Zhang S., Jiang X., Zhang W., Meng F., Gao J., Cheng X., Hu Y., Liu J., Zhao T., Zhu L. (2025). Hypobaric hypoxia exposure impairs colonic goblet cell subpopulation via the HIF-1α signaling pathway. Am. J. Physiol. Gastrointest. Liver Physiol..

[57] Ma Q., Ma J., Cui J., Zhang C., Li Y., Liu J., Xie K., Luo E., Tang C., Zhai M. (2023). Oxygen enrichment protects against intestinal damage and gut microbiota disturbance in rats exposed to acute high-altitude hypoxia. Front. Microbiol..

[58] Moszyńska A., Jaśkiewicz M., Serocki M., Cabaj A., Crossman D.K., Bartoszewska S., Gebert M., Dąbrowski M., Collawn J.F., Bartoszewski R. (2022). The hypoxia-induced changes in miRNA-mRNA in RNA-induced silencing complexes and HIF-2 induced miRNAs in human endothelial cells. FASEB J..

[59] Li J., Adams V., Bannam T.L., Miyamoto K., Garcia J.P., Uzal F.A., Rood J.I., McClane B.A. (2013). Toxin plasmids of Clostridium perfringens. Microbiol. Mol. Biol. Rev..

[60] Guo P., Zhang K., Ma X., He P. (2020). Clostridium species as probiotics: Potentials and challenges. J. Anim. Sci. Biotechnol..

[61] Gavzy S.J., Kensiski A., Lee Z.L., Mongodin E.F., Ma B., Bromberg J.S. (2023). Bifidobacterium mechanisms of immune modulation and tolerance. Gut Microbes.

[62] Ramos-Romero S., Santocildes G., Piñol-Piñol D., Rosés C., Pagés T., Hereu M., Amézqueta S., Torrella J.R., Torres J.L., Viscor G. (2020). Implication of gut microbiota in the physiology of rats intermittently exposed to cold and hypobaric hypoxia. PLoS ONE.

[63] Smith P.M., Howitt M.R., Panikov N., Michaud M., Gallini C.A., Bohlooly-Y M., Glickman J.N., Garrett W.S. (2013). The microbial metabolites, short-chain fatty acids, regulate colonic Treg cell homeostasis. Science.

